# Unravelling the dengue surge in South Asia during 2000–2023: pattern, trend, genomics, and key determinants

**DOI:** 10.1017/S0950268826101095

**Published:** 2026-02-06

**Authors:** Md Asaduzzaman, Mohammad Nayeem Hasan, Md Abdullah Omar Nasif, Dilini Mataraarachchi, Ehsan Ahmed Larik, Joshua Onyango, Masum Billah, Avinash Sharma, Mahbubur Rahman, Farhana Haque, Danai Papakonstantinou, Priyamvada Paudyal, Najmul Haider

**Affiliations:** 1 Department of Engineering, School of Digital, Technology, Innovation and Business,University of Staffordshire, Stoke-on-Trent ST4 2DE, United Kingdom; 2 School of Public Health, University of Memphis, TN 38152, USA; 3 Institute of Epidemiology, Disease Control and Research, Mohakhali, Dhaka, Bangladesh; 4 Ministry of Health, Rev. Baddegama Wimalawansa Thero Mawatha, Colombo 10, Sri Lanka; 5 FELTP Health Department Government of Balochistan, Pakistan; 6 Harper-Keele Veterinary School, Keele University, Staffordshire, ST5 5BG, United Kingdom; 7 BRIC-National Centre for Cell Science, India; 8 UK Public Health Rapid Support Team, Department of Infectious Disease Epidemiology and Dynamics, London School of Hygiene and Tropical Medicine (LSHTM), London WC1E 7HT, United Kingdom; 9 Institute for Global Health and Wellbeing, School of Medicine, Keele University, Staffordshire, ST5 5BG, United Kingdom; 10 School of Life Sciences, Faculty of Natural Sciences, Keele University, Staffordshire, ST5 5BG, United Kingdom

**Keywords:** climate variability, dengue cases, dengue mortality, DENV serotypes, generalized linear model, South Asia, time series analysis

## Abstract

This study aimed to analyse the patterns, trends, genomic characteristics, and key determinants of dengue virus (DENV) infections and associated deaths in South Asia from 2000 to 2023. We collected data from the World Health Organization dengue surveillance dashboard and the health ministries of respective countries, and publicly available data from eight South Asian (SA) countries. Descriptive measures, data visualization techniques, and time series analysis were used to identify key patterns and trends of DENV. A time-scaled phylogenetic analysis was carried out to explore the genomic epidemiology and evolution of DENV. A generalized linear model (GLM) was fitted to identify climatic, demographic, and socioeconomic factors. Between 2000 and 2023, SA countries showed a sharp increase in dengue cases and deaths, contributing to 6.5% of cases and 11.07% of deaths caused by DENV globally. The total cases in the region are projected to grow by approximately 40%, and total deaths by 61% by 2033. The predominant genotypes were DENV2/II, DENV3/I, and DENV3/III. GLM underscores climatic, demographic, and socioeconomic factors associated with DENV infection and deaths. The findings urge intensified public health measures emphasising the need for comprehensive interventions, including vector control, climate adaptation, and strengthened healthcare systems to de-escalate the situation.

## Introduction

Dengue fever, a mosquito-borne illness, is caused by the dengue virus (DENV1–DENV4) within the Flaviviridae family. Transmission to humans most commonly occurs through the bites of *Aedes aegypti (L.)* and *Aedes albopictus* (Skuse) mosquitoes [1]. Currently, DENV is endemic in over 125 countries, and reported cases to the World Health Organization (WHO) have been escalating annually [2]. The disease is characterized by symptoms ranging from mild febrile illness to severe dengue, which can result in life-threatening complications [3]. While the majority of infections (>80%) exhibit no or mild symptoms, leading to lifelong immunity against the specific serotype, reinfection with different serotypes, termed as secondary or tertiary dengue infection, poses a significant risk of severe dengue, potentially culminating in fatal outcomes. In 2024, there were over 14 million cases and 9,000 deaths globally due to DENV infection, marking historic milestones for the virus [1–2].

Dengue fever has emerged as a critical public health challenge in South Asia (SA) with increased incidence over the past five decades, transitioning from sporadic outbreaks to a hyperendemic state in many countries, placing immense pressure on healthcare systems and economies [4]. South Asia, which includes eight countries (Afghanistan, Bangladesh, Bhutan, India, Maldives, Nepal, Pakistan, and Sri Lanka) comprises 3% of the global land area and 21% of the world’s population, has witnessed a rapid increase in the frequency, magnitude, and geographical spread of dengue outbreaks [2]. The region is affected by dengue and other infectious diseases with varying rates [5]. India alone accounts for a significant proportion of the global dengue burden, with official reports often underestimating the actual case load due to limited diagnostic capacities and underreporting [5, 6]. In Sri Lanka, dengue has become the leading cause of hospital admissions during peak seasons [5, 6]. Bangladesh reported more than 25% (n = 1,705) of total global deaths in a calendar year in 2023 [2]. Similar trends were observed in Bangladesh and Pakistan, where seasonal outbreaks often overwhelm the countries’ public health systems [5, 6].

Over the past two decades, dengue has intensified across South Asia, driven by a combination of climatic, demographic, and structural factors. Long-term national analyses from Bangladesh document a sustained increase in dengue incidence since the early 2000s, with strong seasonal dependence on temperature and rainfall and a marked escalation in disease severity in recent outbreaks [2, 7, 8]. India, which accounts for a substantial proportion of the regional burden, has experienced recurrent large-scale epidemics, expanding transmission into peri-urban and rural settings, and the persistent underreporting linked to heterogeneous surveillance and lack of diagnostic capacity [5]. In Sri Lanka, dengue has become one of the leading causes of hospital admissions during epidemic periods, with evidence of hyperendemic transmission and periodic serotype shifts associated with increased risk of severe disease [4]. In Nepal, dengue previously confined to lowland Terai districts has expanded into highland regions, with studies demonstrating strong associations between temperature rise, urbanization, and increasing outbreak frequency, highlighting the role of climate change in facilitating the geographic spread [9, 10]. Regional modelling studies further demonstrate that rising temperatures and altered precipitation patterns have lengthened transmission seasons and enabled dengue emergence in previously cooler areas of South Asia [6]. Genomic and phylogenetic investigations across Asia reveal frequent serotype turnover, regional clustering of genotypes, and increasing cross-border viral movement driven by urbanization and population mobility [11]. Despite these advances, existing studies remain largely country-specific or thematically fragmented, underscoring the need for integrated regional analyses that jointly examine epidemiological trends, genomic diversity, and key climatic and socioeconomic determinants across South Asia.

The temporal and spatial patterns of dengue are closely tied to climatic and ecological factors. Dengue transmission typically peaks during and immediately after the monsoon season, when waterlogged environments create abundant breeding sites for mosquitoes [7, 12]. In recent years, the geographic range of dengue has expanded to previously unaffected regions, including cooler highland areas, facilitated by global warming and urbanization. The rising incidence reflects not only improved reporting but also an increase in transmission intensity.

Several interconnected factors drive dengue’s endemicity in South Asia. These countries share porous border, and the exchanges of travellers increase the risk for regional circulation of infectious diseases including DENV. Environmental factors, particularly the tropical and subtropical climates of the region, provide favourable conditions for the *Aedes* mosquito to thrive. High temperatures and heavy rainfall during monsoons lead to widespread mosquito breeding. Climate change exacerbates this issue by prolonging transmission seasons and expanding the vector’s range into non-endemic areas [13]. Unplanned urbanization, rapid population growth, and urban sprawl have led to overcrowded cities with inadequate infrastructure. Poor waste management and water storage practices in these urban settings create ideal mosquito breeding sites. The mobility of human populations, through international travel and trade, also contributes to introducing and circulating dengue serotypes across borders [14]. The major knowledge gaps are primarily related to incomplete surveillance, trends of cases by countries and in the regions, limited genomic data, and insufficient understanding of environmental and socioeconomic determinants. Additionally, inconsistencies in case definitions and diagnostic capacities across South Asian nations hinder comparative trend analysis [2, 5].

Genomic studies of DENV in South Asia remain sparse, with significant gaps in sequencing data for circulating serotypes and genotypes. While all four DENV serotypes (DENV-1 to DENV-4) have been reported in the region, the dynamics of serotype shifts, viral evolution, and their association with outbreak severity remain poorly understood. The emergence of new viral lineages and their potential impact on vaccine efficacy and disease severity require further genomic surveillance [4, 5]. In this study, we aimed to characterise epidemiological trends and serotypes and understand the factors associated with the geographical distribution of dengue cases and deaths in South Asia from 2000 to 2023.

## Methods

### Overview of data

The study focused on dengue cases and deaths across eight South Asian countries: Afghanistan, Bangladesh, Bhutan, India, Maldives, Nepal, Pakistan, and Sri Lanka. Data were collected from multiple publicly available repositories. We collected annual dengue cases and deaths data from the WHO’s dashboard, the Ministry of Health’s official database and published data of these countries from 2000 to 2023. In some cases, we approached Ministry of Health directly to obtain the annual data (e.g., Sri Lanka). Climatic data, including temperature and rainfall, as well as population data, demographic, and socioeconomic indicators were retrieved from the World Bank Databank [15]. The main outcomes of interest in this study were dengue cases and dengue deaths as per the definition provided by the Centers for Disease Control and Prevention (CDC) [16]. In this study, we used two crucial climatic indicators: mean temperature and total rainfall, several population, health, and demographic indicators: population density, proportion of population with obesity, median age, and the key socioeconomic and development indicator – Gross Domestic Product (GDP) per capita. The mean temperature was used as one of the primary climatic predictors as it captures cumulative thermal exposure relevant to vector survival and viral replication and is commonly used in population-level time-series analyses. All data used were on an annual scale. For phylogenetic analysis, dengue whole genome sequence data were retrieved from the Global Initiative of Sharing All Influenza Data (GISAID) database [17].

### Phylogenetic analysis

High-coverage, complete whole-genome sequences of DENV-1 to DENV-4 were retrieved from the GISAID database [17], selected based on geographic location (Bangladesh, Bhutan, India, Maldives, Nepal, Pakistan, and Sri Lanka), and collection dates spanning from January 1, 2000, to December 31, 2023. Separate time-resolved maximum likelihood (ML) phylogenetic trees (pan-serotype and serotype-specific) were constructed, refined, and annotated using the Nextstrain tool Augur [18–22]. The final dataset included 538 genomes: 99 from DENV-1, 186 from DENV-2, 212 from DENV-3, and 41 from DENV-4. Clades and lineages were assigned using Nextclade [18]. The trees were exported and visualized using Auspice [18].

### Statistical analysis and modelling

We provide summary statistics of cases and deaths per million population including mean, standard deviation/inter-quartile range and confidence intervals of dengue cases and deaths across South Asian countries. We generated plots to demonstrate exploratory insights from the datasets. We employed time-series analysis to identify the most suitable model for forecasting dengue cases and deaths for the next 10 years. To identify the influence of the climatic, population, health, demographic, and socioeconomic indicators, a GLM with Poisson response and log link function was fitted. The GLM for modelling disease cases or deaths took the following form:

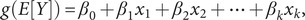

where 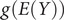 was the link function, 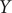 was the response (cases or deaths), and 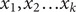 were the predictors. All statistical analyses and model fitting were performed using R version 4.4.1 [23].

## Results

### Dengue cases and deaths in South Asia

**Figure 1.**
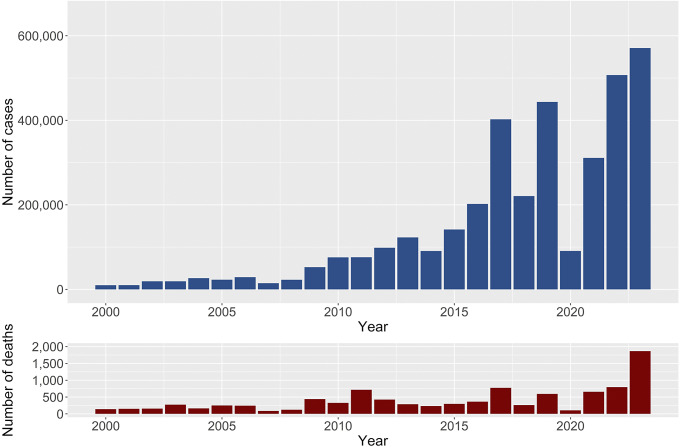
Total number of dengue cases and deaths from 2000 to 2023 for South Asian countries (Afghanistan, Bangladesh, Bhutan, India, Maldives, Nepal, Pakistan and Sri Lanka).

Between 2000 and 2023, South Asia reported a total of 3,581,126 dengue cases and 9,738 deaths, resulting in a case-fatality rate of 0.27%. Compared to 2000–2010, dengue cases increased by 11-fold, while deaths increased by three-fold during 2011–2023. However, the CFR was reduced by 3.5 times between 2000–2010 and 2011–2023. On average, South Asian countries contributed to 6.50% (95% CI: 6.49%–6.51%) of the global cases and 11.07% (95% CI: 10.86%–11.28%) of global deaths due to dengue during the period 2000–2023. Between 2000 and 2023, the number of cases and fatalities showed a consistent upward trend, with significant spikes in later years (Figure 1). By 2023, the region experienced a staggering 570,957 cases and 1,865 deaths-the highest figures recorded in the dataset. Dengue cases and deaths in South Asia exhibited significant variation across the countries from 2000 to 2023. On average, the region reported 18,652 cases and 51 deaths annually, with substantial variability (SD = 43,575 for cases and 145 for deaths) (Table 1).

**Table 1.** Country-wise mean number of dengue cases and deaths per million population and Case-fatality rate of DENV in South Asia over 2000–2023

	Cases/1,000,000		Deaths/1,000,000		Case-fatality rate
SA countries	Mean (SD)	IQR (95% CI)	Mean (SD)	IQR (95% CI)	(%)
Afghanistan	4 (10)	0 (0–8)	0 (0)	0 (0–0)	0.026
Bangladesh	140 (392)	37 (0–305)	0.64 (2)	0.35 (0–1.48)	0.452
Bhutan	608 (1,174)	718 (112–1,103)	0.45 (1.68)	0 (0–1.16)	0.077
India	47 (50)	64 (26–68)	0.12 (0.07)	0.11 (0–0.15)	0.242
Maldives	2,595 (3,759)	4,273 (1,007–4,182)	0.85 (3.06)	0 (0–2.14)	0.031
Nepal	182 (492)	29 (0–390)	0.18 (0.59)	0.05 (0–0.43)	0.096
Pakistan	67 (96)	64 (27–107)	0.15 (0.39)	0.10 (0–0.32)	0.217
Sri Lanka	1,850 (1,899)	1,753 (1,049–2,652)	5.09 (4.83)	3.13 (0–7.13)	0.268
Total	687 (1,791)	245 (432–941)	0.93 (2.72)	0.18 (0–1.32)	0.27

**Figure 2.**
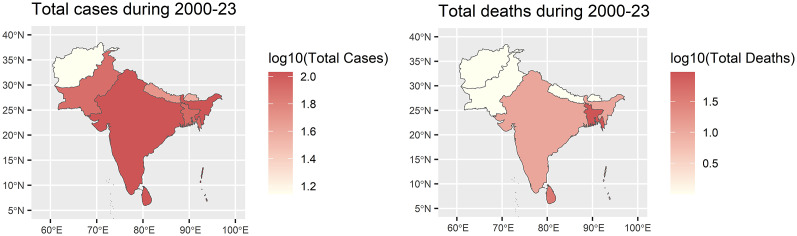
Maps of South Asia showing total dengue cases and deaths in South Asian countries over 2000–2023. A logarithmic scale of 10 is used for data visualization.

When adjusted per million population, the average incidence of dengue cases ranged widely, with the Maldives (2,595 cases per million) and Sri Lanka (1,850 cases per million) indicating the highest rates, while Afghanistan reported the lowest at four cases per million. Sri Lanka also recorded the highest death rate per million population (5.09), with most other countries reporting less than one death per million. Bangladesh reported the highest CFR (0.45%), followed by Sri Lanka (0.27%) and India (0.24%). These trends underscore significant geographical and demographic heterogeneity in dengue burden across South Asia (Figure 2).

### Serotype dominance

**Figure 3.**
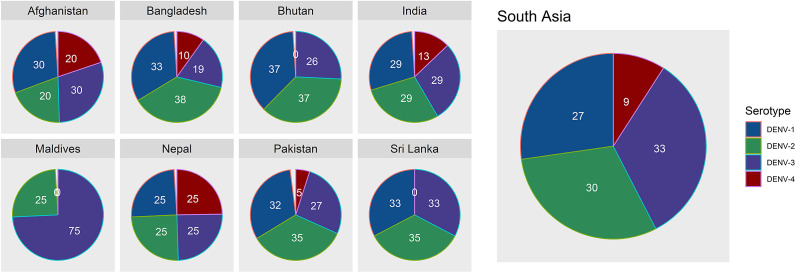
Percentage of dominant dengue virus serotypes in South Asia over 2020–2023.

The percentage of dominant dengue virus (DENV) serotypes in South Asia exhibited significant geographical variation, reflecting the complex dynamics of dengue transmission. DENV-1 and DENV-2 were widely distributed, with dominance percentages ranging from 25% to 37% across most countries over the last 24 years (Figure 3). Overall, serotype 1 dominated the South Asian region (33%), followed by serotype 2 (27%), serotype 3 (30%), and serotype 4 (9%).

### Phylogenetic analysis

**Figure 4.**
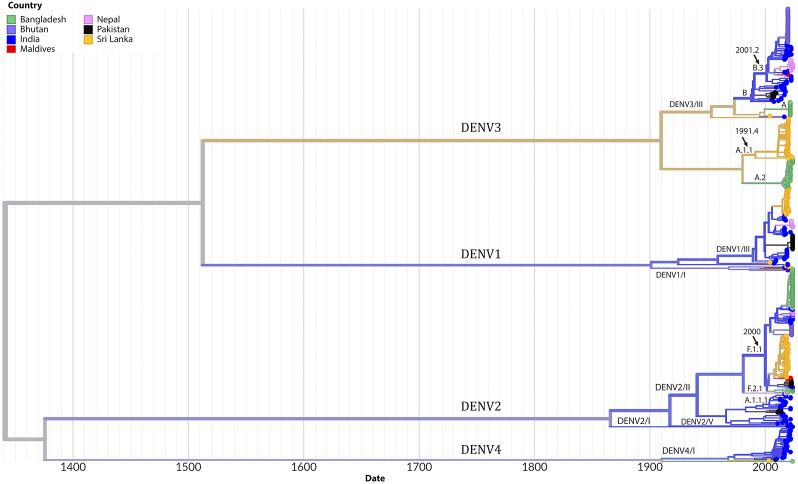
Time-scaled phylogeny of Dengue viruses circulating in South Asia showing 538 genomes sampled between 2003 and 2023. The colour of the tips indicated the host country of the taxa. Branch colour indicated the inferred ancestral geographic location of the descendants. Clades and lineages are indicated adjacent to the key branches. The number above the black arrow denoted the inferred year of introduction of the major lineages in the circulation. Numbers in the x-axis represent the time in years.

**Figure 5.**
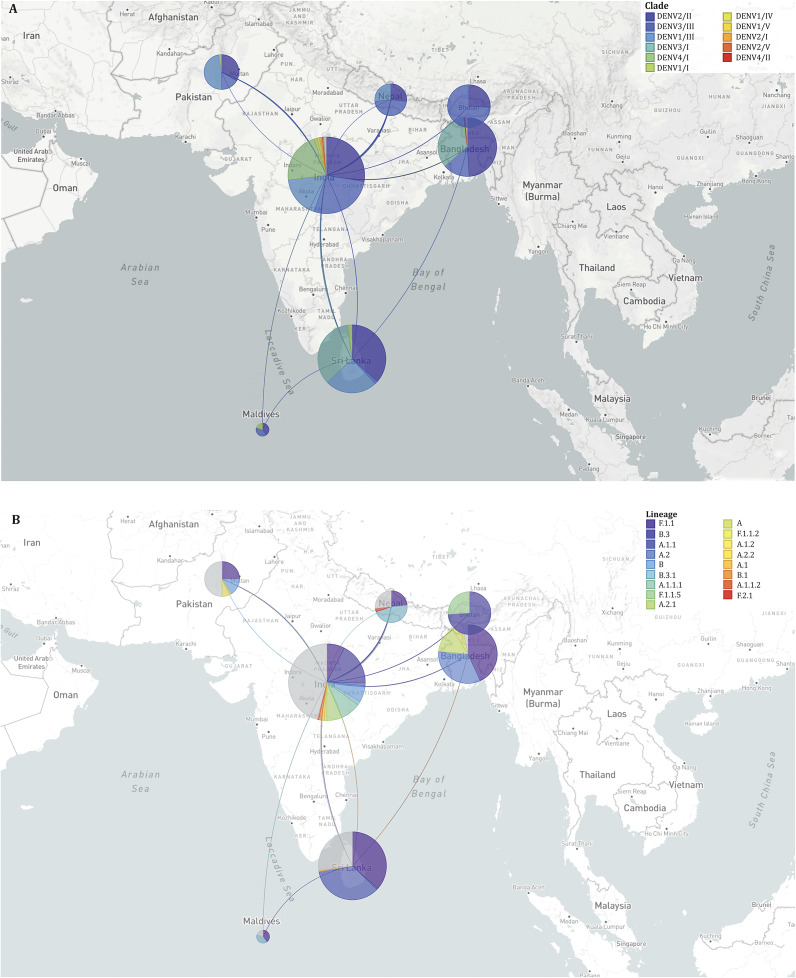
Geographical transmission map of Dengue viruses in South Asia showing the regional movement of viruses at clade (A) and lineage (B) levels. The placement of the coloured circles (demes) in the map is according to the sampling location. The size of the demes indicates the number of sequences sampled from a specific country. The shape of the lines among the demes denotes the direction of the virus movement. Clades and lineages are marked with the respective colour indicated in the legend.

Phylogenetic analysis revealed the circulation of multiple clades for each serotype. Three clades of DENV2 were circulating in SA, among which clade DENV2/II predominated across all seven countries. Several lineages and sub-lineages of DENV2/II were circulating in these countries. The F.1.1 lineage and its sub-lineages F.1.1.2 and F.1.1.5 were prevalent in Bangladesh, Bhutan, India, Nepal, Pakistan, and Sri Lanka (Figure 4). In the case of DENV3, clade DENV3/I and its lineage A.1.1 and A.2 was predominantly circulating in Bangladesh and Sri Lanka whereas clade DENV3/III was circulating in Bangladesh, Bhutan, India, Maldives, and Pakistan. Clade DENV1/III was predominant among the clades of DENV1 circulating in India, Nepal, Pakistan, and Sri Lanka. Clades of DENV4, DENV4/I dominated and circulated in India, Pakistan, and Sri Lanka. The estimated rate of evolution (expressed as substitutions per site per year) was 4.54 × 10^−4^ for all serotypes and stood at 6.67 × 10^−4^ for DENV1, 8.37 × 10^−4^ for DENV2, 4.22 × 10^−4^ for DENV3 and 7.96 × 10^−4^ for DENV4 (Supplementary Figures 2–6). The highest diversity of viruses at the level of clades and lineages was found in India as evident from the transmission map (Figure 5). The transmission map also revealed several events of the movement of viruses among the countries in SA, especially to and from India. At the country level, the majority of sequences analysed originated from India (174) and Sri Lanka (137), while the Maldives contributed the fewest sequences (5). There was no sequence available for Afghanistan in the databases.

### Time series forecasting

**Figure 6.**
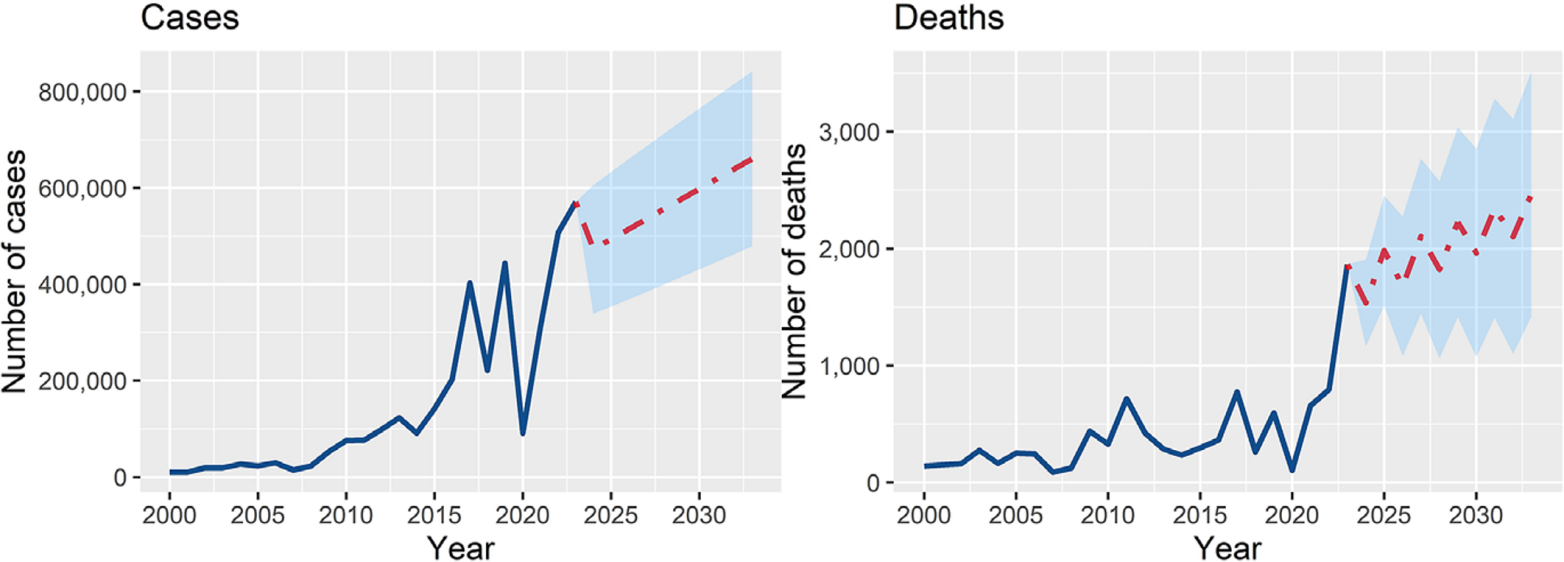
Time series forecasting for cases and deaths in South Asian countries for the period 2024–2033. The model was trained with the data of dengue cases and deaths in the South Asian region for the period 2000–2023.

The observed and forecasted dengue cases and deaths in South Asia from 2000 to 2033 illustrate an upward trend. Total cases are projected to grow from 472,519 in 2024 to 660,609 by 2033, an approximate 40% increase. Similarly, total deaths are expected to rise from 1,535 in 2024 to 2,467 by 2033 (60.7% increase), marking a worrying trend of more severe disease outcomes (Figure 6). Fluctuations in annual values suggest the impact of varying climatic, demographic, and socioeconomic factors, but the overall trend remains upward.

### GLM model results

#### Model for cases

The generalized linear regression model identified key predictors of dengue incidence, expressed as incidence rate ratios (IRR). Population density had an IRR of 1.0138 (95% CI: 1.0137–1.0139, *p* < 0.001), indicating that for every unit increase in population density, the rate of dengue cases increases by 1.38%. Annual average temperature had a notable impact, with an IRR of 1.0942 (95% CI: 1.0897–1.0987, *p* < 0.001), implying a 9.42% increase in dengue cases for every degree increase in temperature. These findings highlight the multifactorial nature of dengue transmission, emphasizing the importance of demographic, and climatic factors in disease dynamics.

#### Model for deaths

The median age, prevalence of obesity among adults, population density, GDP per capita, and dependency ratio were positive and highly significant (*p* < 0.001), suggesting a strong association with dengue fatality. Median age had an IRR of 1.2179 (95% CI: 1.0652–1.3916), indicating a 21.8% increase in the fatality rate for each unit increase in median age, reflecting higher vulnerability in older populations. Prevalence of obesity showed a significant and strong association with fatalities, with an IRR of 2.6910 (95% CI: 2.1895–3.3081) (Table 2).

**Table 2.** Factors affecting the annual incidence of dengue cases and deaths obtained in the fitted generalised linear model (GLM) for South Asian countries, 2000–2023

Dengue cases		Dengue deaths	
Variables	Incidence rate ratio (IRR) (95% CI)	Variables	Incidence rate ratio (IRR) (95% CI)
Population density	1.0138 (1.0137, 1.0139)	Median age	1.2179 (1.0652, 1.3916))
GDP per capita	1.0001 (1.0000, 1.0001)	Prevalence of obesity	2.6910 (2.1895, 3.3081)
Annual average temperature	1.0942 (1.0897, 1.0987)	Population density	1.0233 (1.0220, 1.0246)
Annual total rainfall	0.9995 (0.9995, 0.9995)	GDP per capita	1.0004 (1.0003, 1.0004)
		Dependency ratio	<0.001 (0.000, 0.0001)

*Note:* These variables were adjusted for population size, countries, and years for both models. We presented the values up to 4 decimal points to show the impact of the predictors with tighter confidence intervals.

Both models explain good levels of the deviance (>78% and > 88%), indicating excellent model fit and predictive accuracy. Overall, the GLM models highlight the interplay between climatic, demographic, and socioeconomic variables in influencing dengue cases and deaths in South Asia, providing valuable insights for targeted public health interventions.

## Discussion

Our study highlights the alarming patterns, trends, and multifactorial determinants of dengue outbreaks in the region. The study estimated that South Asian countries contribute 6.5% of cases and 11.07% of deaths to the global dengue burden. Compared to the first decade (2000–2010), mean annual dengue cases and deaths has increased by 11-fold and 3-fold, respectively, although CFR has decreased by 3.5% (during 2011–2023). This study further predicted an increase of dengue cases by 40% and deaths by 61% in the region by 2033. We identified that serotype 1 and 2 and genotype DENV2/II, DENV3/I and DENV3/III were predominantly circulating in the region. Country-wise, Sri Lanka and Maldives recorded the highest incidence (5.09 and 0.85 per million people, respectively), while Banagldesh and Sri Lanka reported highest CFRs (0.45% and 0. 27%, respectively).

South Asia has transitioned from sporadic dengue outbreaks to a hyperendemic state, characterized by the co-circulation of multiple dengue virus serotypes. This is possibly related to secondary cases, which can be associated with more severe disease and increasing number of deaths [8, 24]. Between 2000 and 2023, reported cases surged from under 10,000 to over 570,000 annually, while deaths rose from 140 to 1,865. Countries like India, Sri Lanka, and Bangladesh have borne the brunt of this burden, with Sri Lanka recording the highest case and mortality rates per million population. Notably, the Maldives exhibited high case rates but relatively lower mortality, likely due to the predominance of one circulating serotype (DENV-3) and therefore, lower risk of severe secondary dengue cases, as well as its smaller centralized population enabling efficient healthcare access.

The geographic distribution of dengue highlights significant variability between South Asian countries, with tropical and subtropical climates facilitating year-round transmission. Seasonal peaks, aligned with monsoon seasons, emphasize the role of waterlogging and stagnant water in creating breeding grounds for the *A. aegypti* and *A. albopictus* mosquitoes. Additionally, the rise in dengue cases in previously unaffected highland regions underscores the expanding ecological niche of these vectors, likely driven by global warming and other factors. The interconnected nature of countries contributes to the exchange of serotypes between countries. For example, DENV-2 caused an epidemic in India in 2022, and it caused an epidemic in Bangladesh in 2023 [24].

The time series forecast emphasises the urgent need for strengthened public health interventions, including vector control, climate adaptation strategies, and enhanced healthcare infrastructure to effectively manage the growing dengue burden. Without comprehensive measures, the increasing incidence and mortality may further strain health systems in the region. The study identifies critical climatic, demographic, and socioeconomic drivers of dengue transmission. Climatic variables, particularly temperature and rainfall, emerged as dominant predictors. High temperatures and increased rainfall create optimal conditions for mosquito breeding, while climate change extends transmission seasons and geographic ranges.

Socioeconomic disparities further compound the dengue crisis. Vulnerable populations in low-income settings often lack awareness, resources, and healthcare access, resulting in delayed treatment and underreporting of cases [23]. Moreover, lifestyle changes, including increased obesity prevalence, were associated with higher dengue susceptibility. Obesity-related health issues may exacerbate dengue severity, complicating disease management.

The phylogeography of dengue in South Asia underscores the complex interplay of circulating serotypes, clades, and lineages, shaped by local viral evolution and the frequent regional exchange of new strains. Certain clades and lineages, such as DENV-1/III, DENV-2/II, DENV-3/III, and DENV-4/I, have gained an evolutionary advantage, dominated the region and remained prevalent across most countries in the region. Historically, locally adapted strains of dengue have tended to persist within specific geographical areas for extended periods. However, increasing interconnectivity and rapid human mobility have facilitated the introduction and establishment of new strains in previously unaffected locations [11]. In South Asia, the presence of developed urban centres, porous borders with neighbouring countries, and robust regional interconnectivity have positioned India as a significant hotspot for frequent viral exchange with the surrounding countries. Notably, the rate of dengue virus evolution in SA, aligns with global trends [11, 25], indicating that the evolutionary pressures driving viral divergence in the region are consistent with those observed worldwide.

By employing robust statistical methodologies, including time series analysis and generalized linear modelling, our study elucidates the complex interplay of climatic, demographic, and socioeconomic factors in increasing this health crisis. The findings underscore the need for comprehensive, multi-sectoral approaches to dengue prevention and control in South Asia. Effective multisectoral interventions must address the root causes of transmission, including climate adaptation measures, urban planning reforms, socioeconomic inequalities, and the wider social determinants of health. Targeted vector control strategies, such as improved waste management and community awareness campaigns, are essential for reducing mosquito breeding sites.

Strengthening healthcare infrastructure, particularly in rural and underserved areas, is critical for early diagnosis and treatment. Enhanced surveillance systems (including genomic surveillance) and research into serotype and clade dynamics can all inform vaccine deployment and outbreak response strategies. Additionally, cross-border collaboration is vital to managing the transnational nature of dengue transmission, particularly given the co-circulation of multiple serotypes [26].

Recent evidence highlights the critical role of entomological shifts in shaping the expanding dengue landscape in South Asia. Climate change has substantially altered the ecological suitability of *A. aegypti* and *A. albopictus*, enabling their establishment in higher altitude and previously cooler regions that were historically unsuitable for sustained transmission [27, 28]. Rising minimum temperatures, warmer winters, and changing precipitation patterns have improved vector survival, accelerated larval development, shortened extrinsic incubation periods, and extended seasonal transmission windows [29, 30]. A growing body of literature documents the altitudinal expansion of dengue vectors and dengue transmission across South Asia and the Hindu Kush Himalayan region, including mid and high-altitude areas of Nepal, Bhutan, northern India, and Pakistan, where dengue was previously rare or absent [27]. Empirical evidence from the Hindu Kush Himalayan region demonstrates that climate warming has increased thermal suitability and vectorial capacity for *Aedes* mosquitoes, facilitating their upward spread along altitudinal gradients and increasing the risk of dengue emergence in mountain communities [31]. In addition, experimental and physiological studies show that temperature driven changes directly influence mosquito development, survival, biting behaviour, and viral replication efficiency, thereby amplifying dengue transmission potential under warming scenarios [29]. Collectively, these entomological findings provide a mechanistic explanation for the observed geographic expansion of dengue into highland regions and reinforce the need to integrate vector surveillance, entomological monitoring, and climate adaptation strategies into dengue prevention and control programmes across South Asia.

This study provides a nuanced understanding of these factors, offering valuable insights for policymakers and public health practitioners. Without urgent and sustained interventions, the upward trajectory of dengue incidence and mortality will continue to strain health systems and disrupt livelihoods across the region.

## Limitations

The main strength of this study lies in its comprehensive multi-dimensional approach, combining long-term epidemiological trend analysis, genomic phylogenetics, and modelling of climatic, demographic, and socioeconomic determinants across eight South Asian countries. This integration provides a holistic understanding of dengue dynamics, offering robust evidence to guide targeted interventions, vaccine strategies, and regional public health policy. However, the reporting of dengue cases and deaths in South Asian countries has several limitations, including inconsistencies in surveillance systems, underreporting, and misclassification. In countries such as India, Bangladesh, Pakistan, Sri Lanka, Nepal, Bhutan, Maldives, and Afghanistan, dengue surveillance is often challenged by inadequate diagnostic facilities, leading to the misidentification of cases as other febrile illnesses or vice versa, where other febrile illnesses with dengue-like features could have been counted as dengue cases. Additionally, many infections likely remain undiagnosed due to a high proportion of asymptomatic or mild cases that do not seek medical attention, resulting in underreporting. Inconsistencies in case definitions and reporting criteria across national surveillance programmes further lead to data discrepancies. Some countries rely on passive surveillance systems, which primarily capture cases from healthcare facilities, missing community-level infections and underestimating the true burden of the disease, especially for Afghanistan, which is also highly likely for Bangladesh. Furthermore, limited access to laboratory confirmation, especially in rural areas, impairs accurate case detection. The underreporting of dengue-related deaths may be exacerbated by misclassification, where deaths due to severe dengue complications, such as organ failure or haemorrhagic manifestations, may be attributed to other causes. Political and economic constraints also influence the robustness of reporting mechanisms, with some countries lacking the resources for continuous epidemiological monitoring. Seasonal variations in dengue transmission and the influence of climate change further complicate reporting efforts, as outbreaks may occur unpredictably and overwhelm health systems. Moreover, differences in data transparency and government priorities affect the availability and reliability of epidemiological data. Regional collaboration and standardized surveillance guidelines from organizations such as the WHO are crucial for improving dengue reporting accuracy. In addition, the modelling framework relied on mean temperature and did not incorporate minimum and maximum temperatures. This may have limited the ability to capture thermal extremes and to fully characterise the uncertainty surrounding temperature-related estimates. Addressing these limitations through enhanced diagnostic capacity, active surveillance, enhanced cross-border collaboration, and public health interventions is necessary to obtain a more accurate assessment of dengue’s burden and to develop effective control strategies in South Asia.

## Supporting information

10.1017/S0950268826101095.sm001 Asaduzzaman et al. supplementary material Asaduzzaman et al. supplementary material

## Data Availability

The findings of the phylogenetic analysis are based on metadata associated with 538 Dengue whole genome sequences available on GISAID up to December 31, 2024, and accessible at https://doi.org/10.55876/gis8.250113xb (Supplementary Table). Dengue cases and deaths were compiled from WHO’s Global Dengue Surveillance Platform, the Ministry of Health websites of several countries, and, in some instances, through personal communication with Ministry of Health officials via the co-authors of this article. The full dataset is available upon request from the corresponding author.

## Supplementary material

The supplementary material for this article can be found at http://doi.org/10.1017/S0950268826101095.
