# Supplementary material for: Unravelling the dengue surge in South Asia during 2000–2023: pattern, trend, genomics, and key determinants
Source: Epidemiol Infect. 2026 Feb 6;154:e28. doi: 10.1017/S0950268826101095 (PMC12964148; doi:10.1017/S0950268826101095)
Supplement: Asaduzzaman et al. supplementary material [file S0950268826101095sup001.docx]

**Unravelling the dengue surge in South Asia during 2000-2023: pattern, trend, genomics, and key determinants**

**Appendix:**


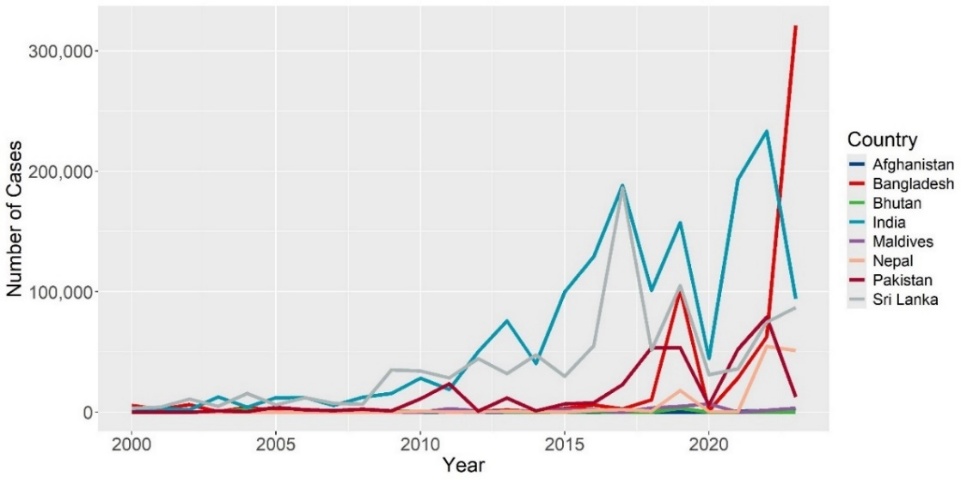


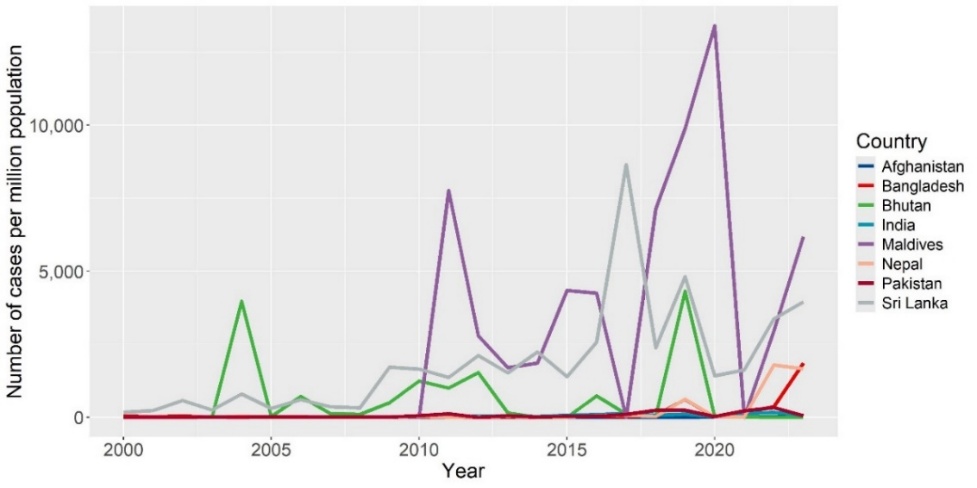


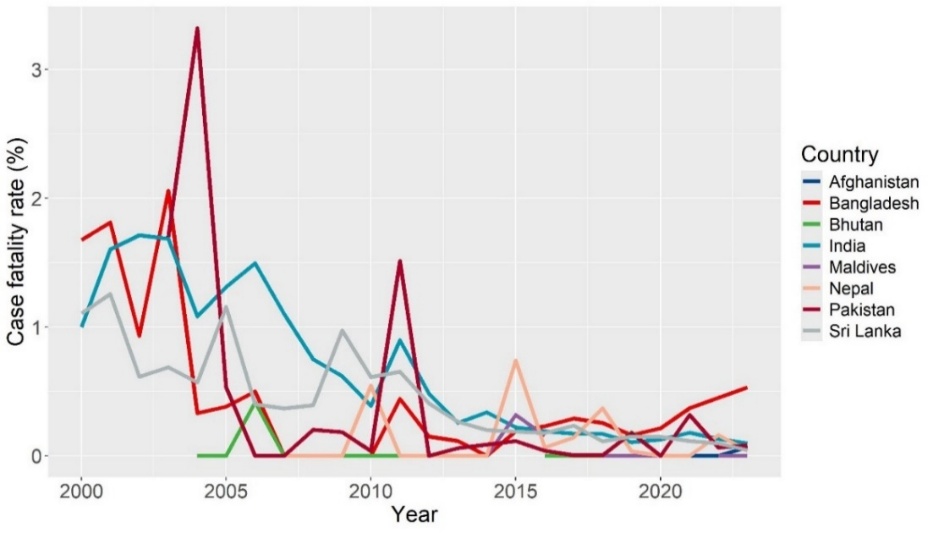


Figure 1: Total number of dengue cases, cases per million and case fatality ratio in SA countries over 2000 to 2023

**
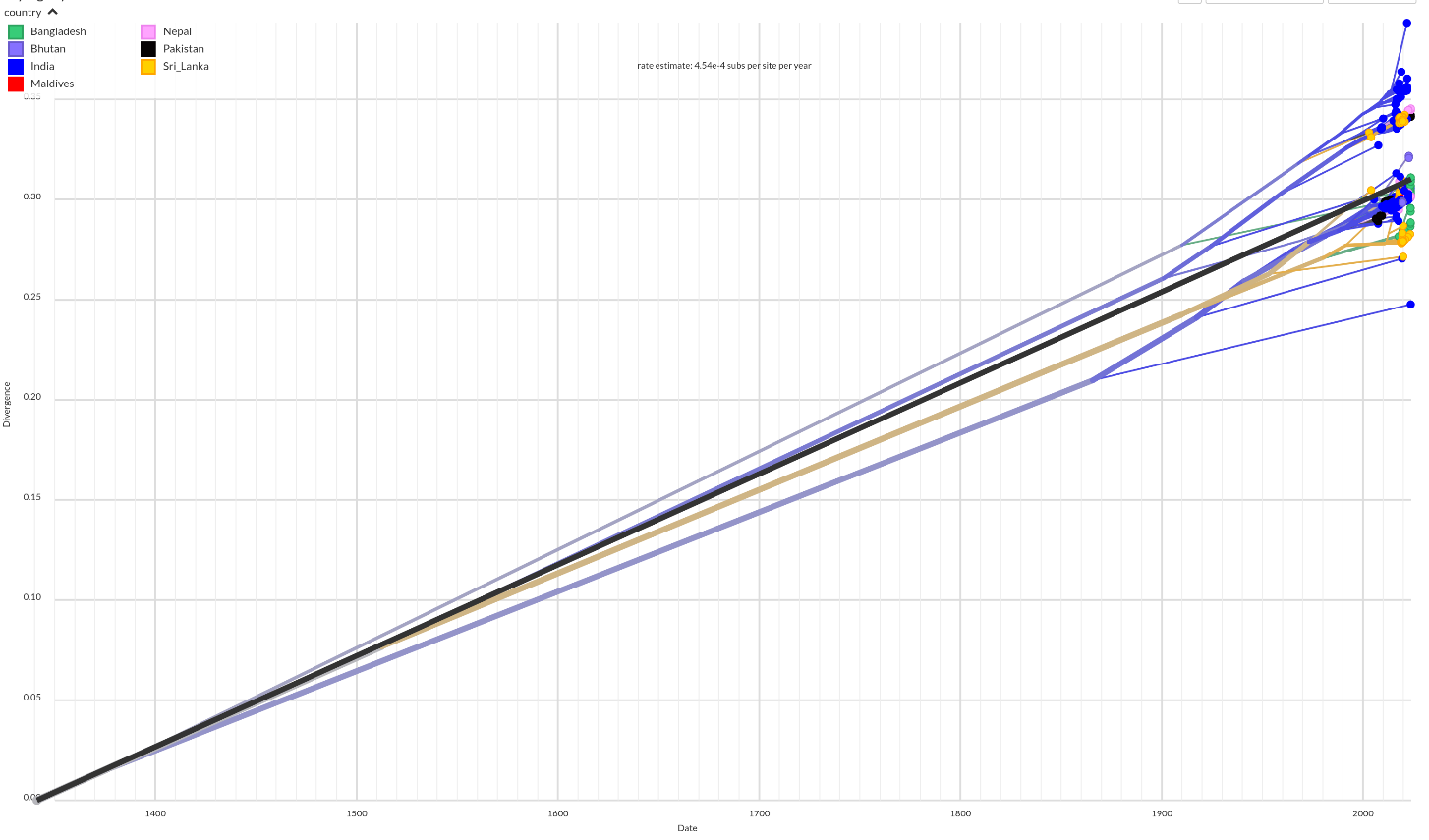
**

**
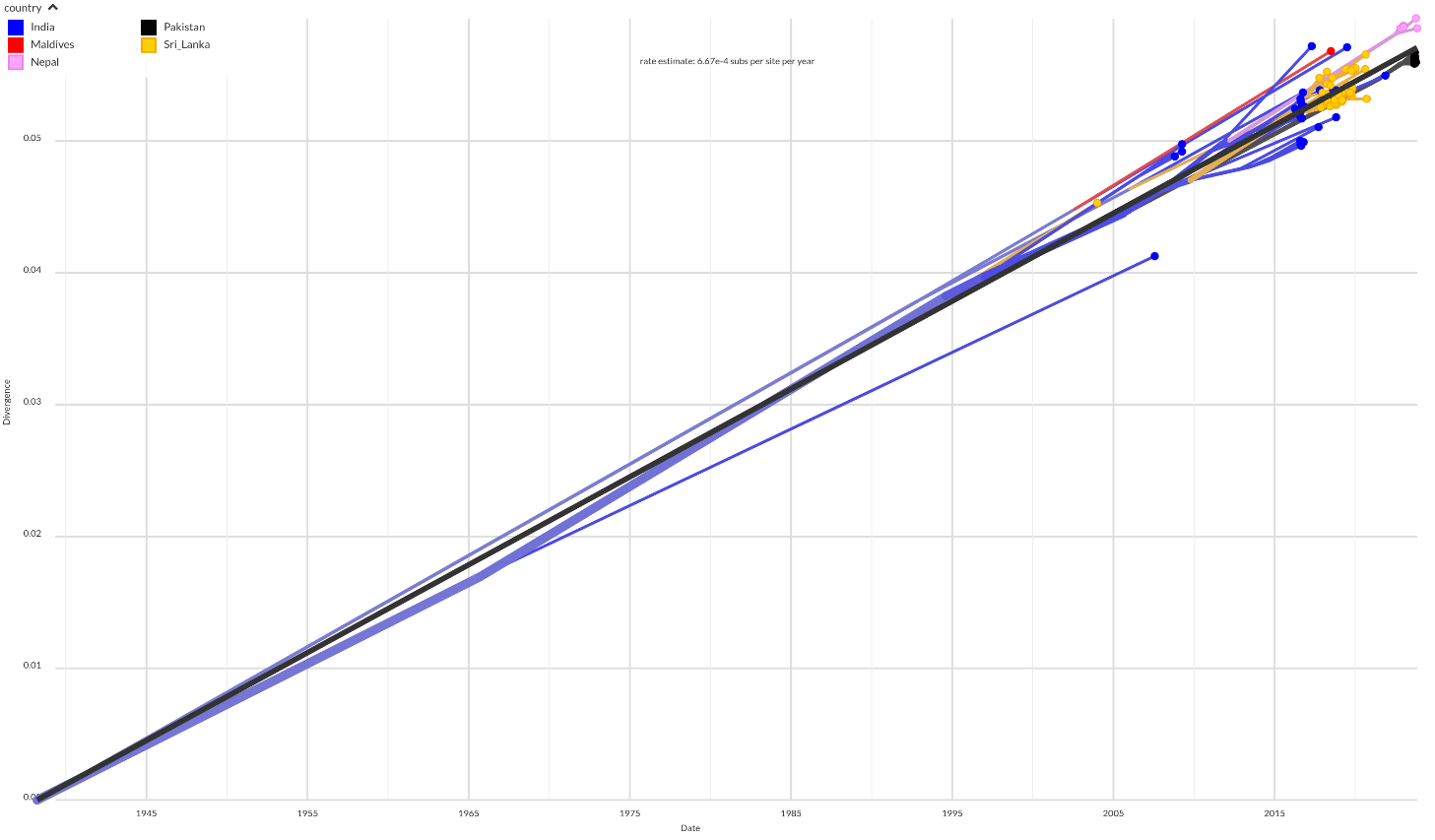
**

**
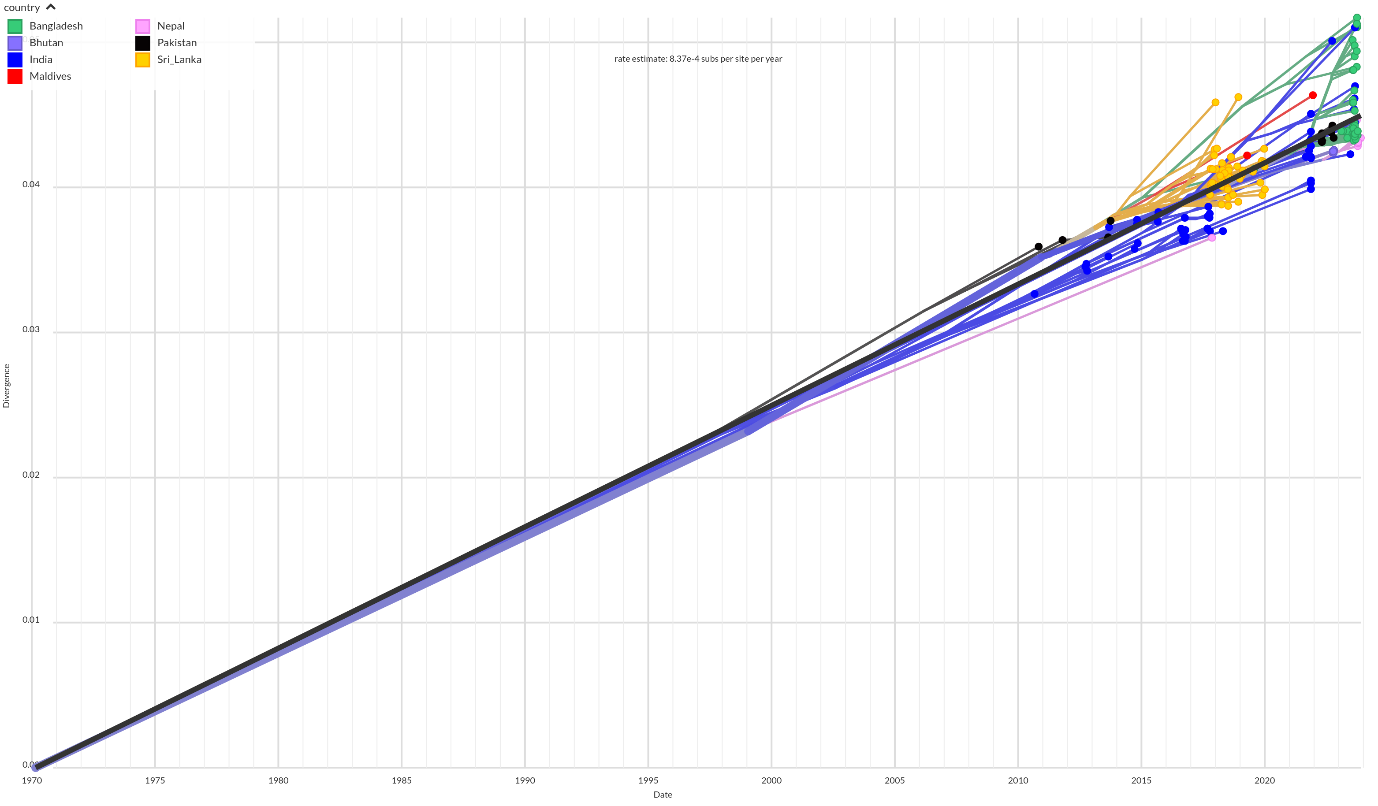
**

**
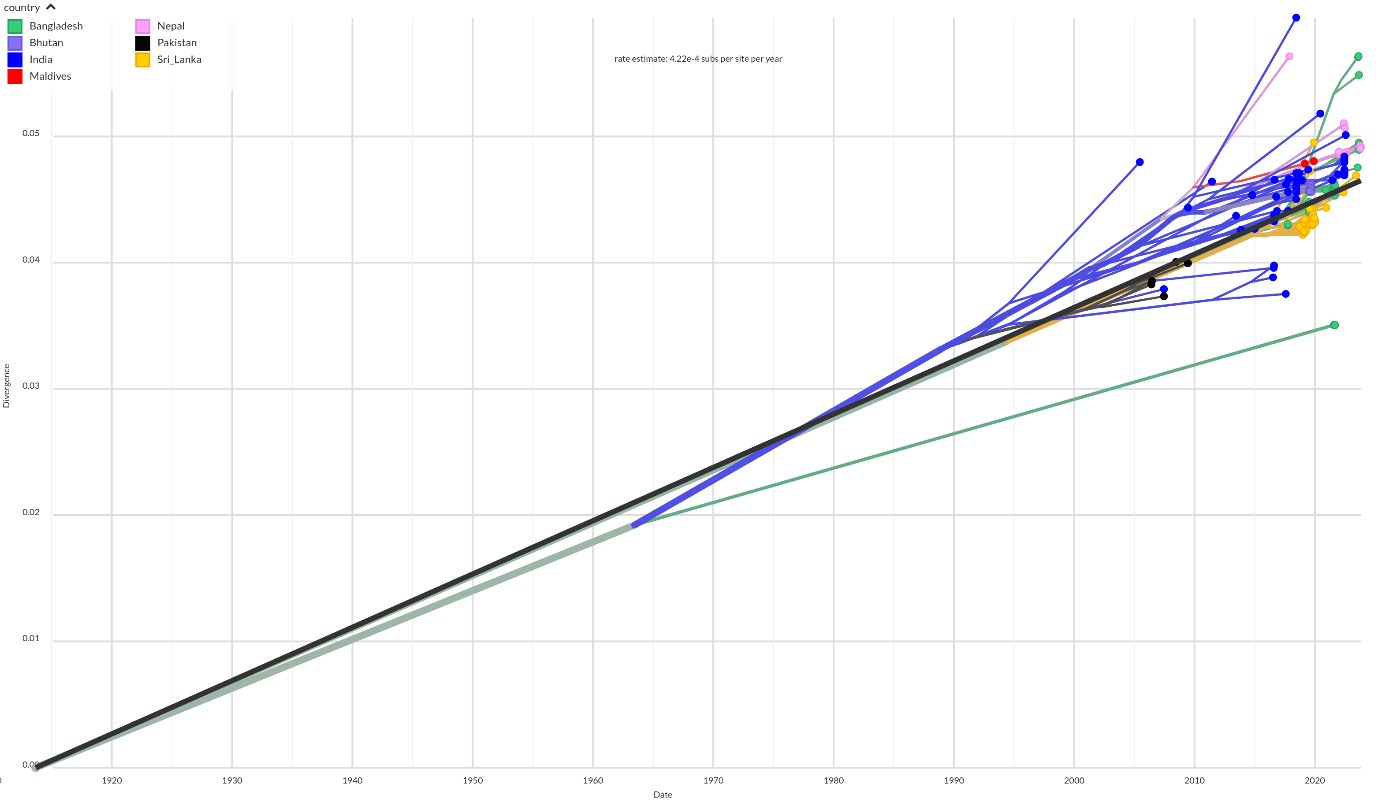
**

**
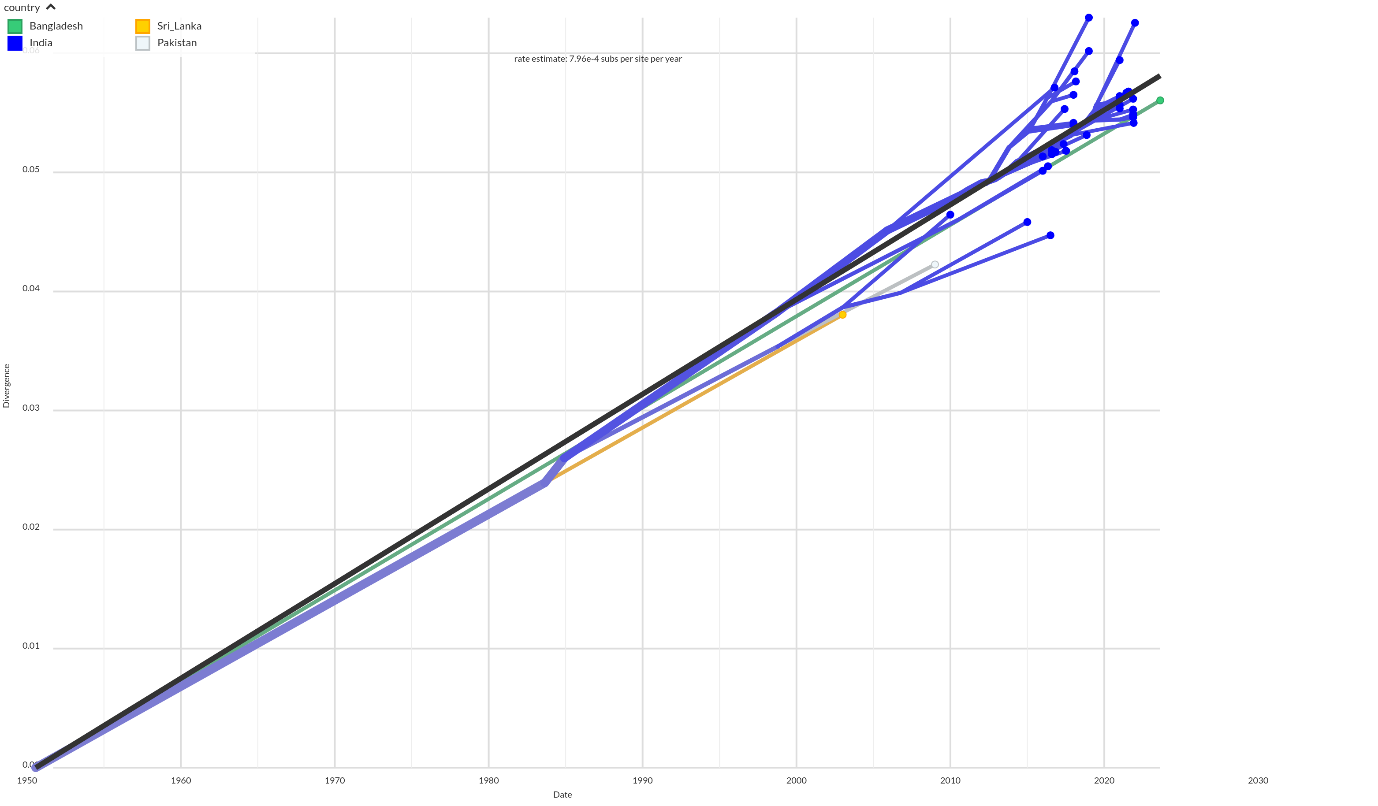
**

Figure 2: Root-to-tip regression analyses DENV (pan-serotype), DEN1, DEN2, DEN3 & DEN4. Plots of the root-to-tip genetic distance against sampling time are shown here. Sampling dates are given as years.
